# Multi-Omic Admission-Based Prognostic Biomarkers Identified by Machine Learning Algorithms Predict Patient Recovery and 30-Day Survival in Trauma Patients

**DOI:** 10.3390/metabo12090774

**Published:** 2022-08-23

**Authors:** Sultan S. Abdelhamid, Jacob Scioscia, Yoram Vodovotz, Junru Wu, Anna Rosengart, Eunseo Sung, Syed Rahman, Robert Voinchet, Jillian Bonaroti, Shimena Li, Jennifer L. Darby, Upendra K. Kar, Matthew D. Neal, Jason Sperry, Jishnu Das, Timothy R. Billiar

**Affiliations:** 1 Department of Surgery, University of Pittsburgh, Pittsburgh, PA 15213, USA; 2 Pittsburgh Trauma and Transfusion Medicine Research Center, University of Pittsburgh, Pittsburgh, PA 15213, USA; 3 Eight-Year Program of Medicine, Xiangya School of Medicine, Central South University, Changsha 410013, China; 4 Center for Systems Immunology, Departments of Immunology and Computational & Systems Biology, University of Pittsburgh, Pittsburgh, PA 15213, USA

**Keywords:** multi-omics, trauma, machine learning, biomarkers, prognostic, proteomics

## Abstract

Admission-based circulating biomarkers for the prediction of outcomes in trauma patients could be useful for clinical decision support. It is unknown which molecular classes of biomolecules can contribute biomarkers to predictive modeling. Here, we analyzed a large multi-omic database of over 8500 markers (proteomics, metabolomics, and lipidomics) to identify prognostic biomarkers in the circulating compartment for adverse outcomes, including mortality and slow recovery, in severely injured trauma patients. Admission plasma samples from patients (*n* = 129) enrolled in the Prehospital Air Medical Plasma (PAMPer) trial were analyzed using mass spectrometry (metabolomics and lipidomics) and aptamer-based (proteomics) assays. Biomarkers were selected via Least Absolute Shrinkage and Selection Operator (LASSO) regression modeling and machine learning analysis. A combination of five proteins from the proteomic layer was best at discriminating resolvers from non-resolvers from critical illness with an Area Under the Receiver Operating Characteristic curve (AUC) of 0.74, while 26 multi-omic features predicted 30-day survival with an AUC of 0.77. Patients with traumatic brain injury as part of their injury complex had a unique subset of features that predicted 30-day survival. Our findings indicate that multi-omic analyses can identify novel admission-based prognostic biomarkers for outcomes in trauma patients. Unique biomarker discovery also has the potential to provide biologic insights.

## 1. Introduction

Trauma is one of the leading causes of human morbidity and mortality worldwide, resulting in both short- and long-term health impairment and a significant economic burden [1]. The clinical trajectories for patients with seemingly similar injury complexes can vary widely and the basis for this variation remains unknown [2,3]. Some patients recover quickly, while others suffer from further complications such as nosocomial infection and sepsis, multiple organ dysfunction, and cognitive disabilities resulting in extended intensive care unit lengths of stay (ICU LOS) [4,5].

Advances in prehospital patient management and inpatient hospital care at designated trauma centers have increased 30-day survival in trauma patients. This improved survival is associated with a concomitant increase in the percentage of patients who remain critically ill after trauma [4,6]. Given its well-defined starting point, trauma as a disease process can be easily studied longitudinally from onset to clinical outcome. However, specific and sensitive biomarkers to predict survival or clinical course at the time of presentation are limited [7,8].

Large-scale, admission-based multi-omic profiling has the potential to identify unique prognostic biomarkers and point to biologic processes associated with patient outcomes. A deep analysis of a high dimensional multi-omic dataset derived from a clinical trial (Prehospital Air Medical Plasma [PAMPer] trial) that demonstrated a survival benefit of prehospital thawed plasma was previously reported [9,10], as well as proteomics from a clinical trial that assessed the use of tranexamic acid after injury (Study of Tranexamic Acid During Air Medical and Ground Prehospital Transport [STAAMP]). Both of these studies were interventional, placebo-controlled, randomized clinical trials. The standard-of-care arm from these multi-institutional studies is representative of severely injured adult patients transported to Level 1 trauma centers in the USA. Temporal and outcome-based patterns seen in severely injured trauma patients were previously analyzed [10,11,12]. Here, we identified which markers have the potential to predict specific outcomes from an admission-based blood draw taken in the emergency department. In order to account for the high-dimensional multi-omic datasets, statistical methods with the capability of dimensionality reduction and feature selection were used to assess the predictive power of these markers. We report here the application of machine learning algorithms to a database of proteomic, lipidomic, and metabolomic plasma markers to identify admission-based prognostic biomarkers for adverse outcomes.

## 2. Methods

### 2.1. Patient Enrollment

Human plasma samples (*n* = 112 for PAMPer and *n* = 93 for STAAMP) were obtained upon emergency department admission (0 h (h) timepoint). Patients transported by air ambulance were enrolled if they had tachycardia (defined as a heartrate > 108 beats per minute) and experienced at least one episode of hypotension (defined as systolic blood pressure < 90 mm Hg) or if they experienced severe hypotension (defined as systolic blood pressure < 70 mm Hg). Patients from the PAMPer dataset were used to train our models, which were validated for proteomics using patients from the STAAMP clinical trial. Further information on enrollment criteria for the PAMPer and STAAMP trials have been published [9,13]. Injury severity score (ISS) was calculated based on the established Abbreviated Injury Scale score that is allocated to one out of six body regions. The 3 scores from the most severely injured body regions produce the ISS score, as described previously [13,14].

### 2.2. IRB Approval

This study was conducted according to the guidelines of the Declaration of Helsinki and approved by the Institutional Review Board (IRB) of the University of Pittsburgh (code NCT01818427, 26 March 2013). The PAMPer Trial received approval through the Emergency Exception from Informed Consent (EFIC) protocol from the Human Research Protection Office of the U.S. Army Medical Research and Material Command. Detailed information and study protocol for the PAMPer Trial are available through the following link: https://clinicaltrials.gov/ct2/show/NCT01818427 (accessed on 1 June 2022).

The Study of Tranexamic Acid During Air Medical and Ground Prehospital Transport (STAAMP) Trial enrolled adult trauma patients that had at least 1 episode of hypotension (systolic blood pressure < 90 mm Hg) or tachycardia (heart rate 110 bpm or greater). This study received approval through the EFIC protocol by the U.S. Food and Drug Administration as well as a community consultation process under the investigational new drug application (IND) protocol (IND # 121102). Detailed information and study protocol for the STAAMP Trial are available through the following link: https://www.ncbi.nlm.nih.gov/pmc/articles/PMC4623322/#S3title (accessed on 1 June 2022).

### 2.3. Untargeted Metabolomic and Lipidomic Assays

Untargeted lipidomic and metabolomic assays (898 metabolites and 997 lipids) were conducted by Metabolon (Durham, NC, USA); detailed information can be found in our previous analysis [10,11]. Briefly, an ultra-high performance liquid chromatography mass spectrometry (UPLC-MS) platform was utilized for the extraction of the raw data. Following this, the data were analyzed and processed by Metabolon through their in-house software and extensive library of purified standard compounds.

### 2.4. Multiplexed Proteomics Assay

An aptamer-based, multiplexed method was used to measure protein levels (7000+ proteins, SomaLogic Inc., Boulder, CO, USA) for 84 patients included in this study. Further details on the specific techniques are found in [15].

### 2.5. Data Normalization, Noise Reduction, and Scaling

Missing data were less than 10% across all variables and the mean value for each variable was used to fill for any missing data points. Other methods of missing data imputation did not affect the data due to the low number of missing data. A prescreening of the biomarkers was conducted to reduce each layer (metabolomic layer, proteomic layer, lipidomic layer) to the same number of variables based on median and quartiles to filter the most variable markers between patient groups. The bottom quartiles were removed based on abundance and the top 10% most variable markers between groups using R were kept. Each omic layer consisted of approximately 550 biomarkers after this prescreening, as shown in Figure S1. These omic databases were then both analyzed as separate data layers and joined as a combined database, and the data were autoscaled (z-score) across each variable using natural logarithmic transformation and scale functions on R. This is important for dimensionality reduction and heatmap graphics. Z-scores for the heatmap were calculated within the ComplexHeatmap package in R. These datasets can be found online [10,15,16]. Data are available in a publicly accessible repository. The data presented in this study are openly available in https://data.mendeley.com/datasets/vt8nhp2y2t/1 (accessed on 1 June 2022).

### 2.6. Feature Selection and Regression Analysis

Several statistical and predictive models (Receiver Operating Characteristic (ROC) Curve, generalized linear model with Least Absolute Shrinkage and Selection Operator (LASSO) regression, support vector machine, random forest) were used to evaluate the predictive nature of biomarkers on either the individual layer scale or on the multi-omic platform for a binary outcome (resolving status, 30-day survival, and 30-day survival in patients with traumatic brain injury (TBI)). The R Caret package was used to train the data with a 10-fold cross validation. A Mann–Whitney U-test was incorporated to rank the significance and contribution of each biomarker during the analysis for both support vector machine and random forest methods of machine learning analysis along with a Benjamini–Hochberg correction with a false discovery rate of less than 5%.

Linear regression with LASSO regularization was tuned by 6 different lambda (penalty) values ranging from 0.5 to 1 by 0.1 increments, making sure only balanced lambda coefficients were chosen to represent the model to avoid over- and under-fitting errors. These regularized data were used to train the support vector machine and random forest models.

### 2.7. Gene Set Enrichment Analysis

GSEA was carried out using the proteins’ EntrezGeneID and the Princeton GO term site (https://go.princeton.edu/cgi-bin/GOTermMapper) (accessed on 1 June 2022).

### 2.8. Predictive Modeling at Admission

The regularized data were used to train two different separate methods of machine learning (support vector machine and random forest). For each outcome tested (resolution from critical illness, 30-day survival, 30-day survival with TBI), these models were repeated 20 times with 10-fold cross validation at each lambda value tested. The performance of each model was examined by calculating both the accuracy and Area Under the ROC Curve (AUC) of each model. The layers (proteomics only, lipidomics only, metabolomics only, and the 3 combined into one multi-omic layer) were analyzed to determine which had the best predictive potential based on highest AUC and accuracy. For 30-day survival and TBI, the multi-omic predictive model functioned best with 84 patients and approximately 1650 biomarkers per run. For prediction of resolving status, the proteomic layer performed best with 84 patients and 550 biomarkers.

### 2.9. Cross-Prediction Validation across Two Trials

To validate the accuracy of the models, proteomic data from the standard-of-care arm patient cohort derived from the STAAMP study [13] were used to validate the performance of the machine learning model for proteomic markers. Data normalization and scaling were applied to the STAAMP proteomic layer in the same manner as outlined above. Based on the results of the PAMPER random forest cross-validated proteomic analysis, the same features (5 total) from this model were filtered for in the STAAMP proteomic data. The random forest machine learning model was trained on these features from PAMPER proteomic data and used to predict the outcome of the STAAMP proteomic data (the STAAMP dataset was used for independent validation).

## 3. Results

### 3.1. Overview of Study Design, Details, and Multi-Omic Layers

**Figure 1:**
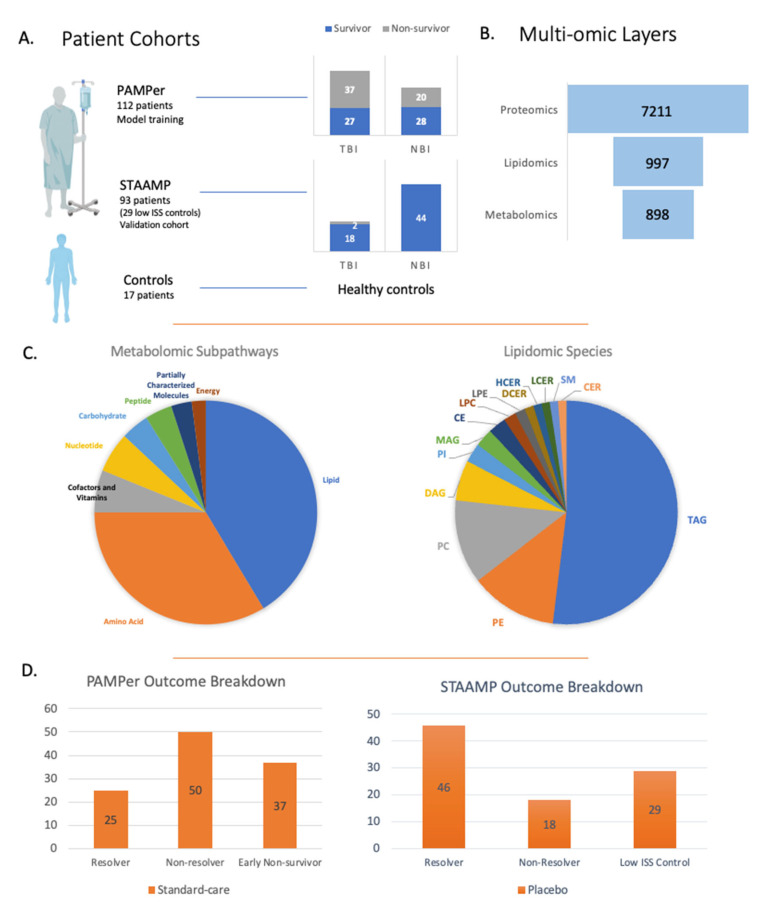
Overview of patient cohorts and multi-omic layers. (**A**) Breakdown of patients from the clinical trials used to develop the predictive model and validation model looking at 30-day survival and traumatic brain injury. (**B**) Breakdown of multi-omic layers and number of variables used for the predictive modeling. (**C**) Metabolomic and lipidomic subpathways and species. (**D**) Overview of patients from PAMPer and STAAMP clinical trials looking at outcome based on ICU LOS/time of death.

The admission circulating multi-omic data from patients in the standard-care arm of the PAMPer Trial (*n* = 112), a prospective trial that enrolled severely injured trauma patients transported to trauma centers via air ambulance [9], were used to identify prognostic biomarkers. The proteomic findings were validated using the standard-care arm from the STAAMP trial (*n* = 93) [13]. The multi-omic datasets are outlined in Figure 1 and in our previous study describing the human response to severe injury [10]. Omic data included untargeted metabolomics (898 metabolites) [10], quantitative lipidomics (987 lipids) [11], and multiplexed assay-based proteomics (7211 proteins) [15].

Patients in the primary cohort were segregated based on three outcomes as outlined in Table 1. In the first analysis, patients were divided into resolvers from critical illness (intensive care unit length of stay (ICU LOS) < 7 days, *n* = 25) vs. non-resolvers (ICU LOS ≥ 7 days and/or died, non-resolvers, *n* = 50). The second clinical outcome was 30-day survival with 55 survivors and 57 non-survivors. Finally, we assessed 30-day survival in patients with TBI (survivors *n* = 27, non-survivors *n* = 28). Non-fasting healthy controls (*n* = 17, 12 males, 5 females, age distribution 20–66 years, with mean age 42.5 years) were included and described in previous metabolomic and lipidomic studies [10,11], while low injury severity score (ISS) trauma patients (*n* = 29, ISS score of 1 or less) were from the STAAMP trial and were used for controls in the proteomic analysis.

### 3.2. Proteomics Predicts Resolution from Critical Illness

First, multivariate machine learning analysis was used to determine which individual data layer provided the most predictive features for outcomes. This was then compared to a multivariate statistical model that combined the data layers of all three omics. LASSO-based feature selection was used to classify the down-selected features. To prevent over-fitting, L1-regularization and cross-validation were performed in every run. L1-regularization was used for this high-dimensional dataset as the number of features was higher than the number of sums. The performance of the models (using AUC) was measured in a k-fold cross-validation framework with permutation testing, as described previously [17,18,19]. The use of cross-validation provides a rigorous way to evaluate predictive performance with data held out. Non-resolvers were defined as patients who had slow recovery with an intensive care unit length of stay of 7 days or longer. The proteomic layer alone was the best at discriminating resolvers from non-resolvers with an AUC of 0.74, as shown in Table 2. The metabolomic and lipidomic layers individually, along with a combination of these layers, yielded AUCs ranging between 0.68 and 0.71 (Table S1).

**Figure 2:**
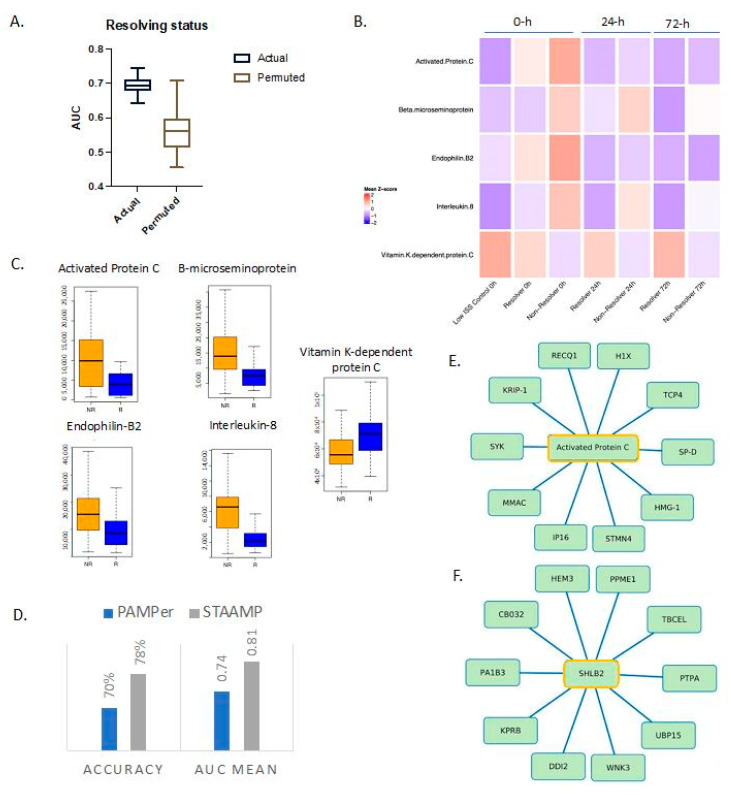
Proteomic markers predict resolution of critical illness based on ICU length of stay and time of death. (**A**) Actual and permuted LASSO AUC graph showing AUC of 0.74 for the levels of 5 proteins. (**B**) Heatmap of selected proteins for different outcomes based on time of death and ICU length of stay over time; 0, 24, and 72 h refer to time from emergency department admission. Low ISS controls *n* = 29, resolvers *n* = 25, non-resolvers *n* = 50. (**C**) Box plots of LASSO-selected features that are significantly different between resolvers and non-resolvers with raw value measurement. (**D**) Selected features from model run on PAMPer patients predict non-resolving status in a validation cohort (STAAMP clinical trial) with an AUC of 0.81 and an accuracy of 78%. (**E**,**F**) Correlation network between selected features and proteomic database at Spearman’s r = 0.70 showing direct associations (selected feature outlined in yellow). SHLB2 = Endophilin-B2.

LASSO identified a minimal set of five proteins (Table S2) that were significantly different between resolvers and non-resolvers at admission. Both activated protein C and vitamin-K-dependent protein C (inactive form) were selected as part of the model. In non-resolving patients, higher quantitative levels of activated protein C were observed, while resolvers had higher inactivated protein C (Figure 2C). Endophilin-B2 (EB2) was also higher in the non-resolving group. The functions of EB2 are not well understood but are known to be related to mitochondrial apoptosis, regulation of growth factor signaling, and autophagy induction [20,21]. Beta-microseminoprotein, a member of the immunoglobulin-binding family, and the chemokine interleukin-8 (IL-8), were both higher in non-resolving patients early after injury. Measured together at admission, these five proteins have the potential to predict recovery from critical illness.

We next characterized the temporal patterns of the five protein biomarkers based on additional sampling time points of 24 and 72 h (Figure 2B). Activated protein C returned to levels seen in low ISS controls by 24 h while the inactive form remained higher in the resolving group through 72 h after injury. Endophilin-B2 also returned to levels similar to resolvers by 24 h. In contrast, beta-microseminoprotein and IL-8 remained consistently higher over time in the non-resolving group compared to resolvers.

To gain a more comprehensive understanding of the entire spectrum of features tracking with outcome, we constructed correlation networks, as described previously, around the LASSO-selected biomarkers [17,18,19]. The most significant associations (Spearman’s r > 0.7) were examined with the full networks for all five biomarkers shown in Figure S2. We also showed focused networks demonstrating correlations involving activated protein C (Figure 2E) and endophilin-B2 (Figure 2F). Proteins in the correlation networks are listed in Table S3.

Activated protein C is an anti-coagulant that degrades the inactivated forms of coagulation factors V and VIII. Activated protein C also has anti-inflammatory and anti-apoptotic properties [22]. Strong, direct associations were found between activated protein C and high mobility group protein B1 (HMGB1). HMGB1 is a well-described damage-associated molecular pattern protein [23,24] known to drive inflammation and organ injury in trauma models [25]. This network also included gamma-interferon-inducible protein 16, which is involved in transcriptional regulation and innate immunity [23]. The correlation network included several other proteins in pathways relating to genotoxic stress resistance, DNA repair, and transcription of zinc-binding domains that regulate gene expression [22].

Endophilin-B2 showed strong associations with ubiquitin carboxyl-terminal hydrolase 15, a key regulator of TGF-β receptor signaling, as well as serine/threonine-protein kinase that plays a role in cell survival via the caspase-3-dependent pathway [23]. Associations were also found with platelet-activating factor acetylhydrolase IB subunit gamma, which functions in brain development and cognitive disability, and a protein regulator of voltage-gated calcium channels on cannabinoid receptors [22,23].

To validate our findings, we carried out a cross-prediction analysis using data from patients in the STAAMP clinical trial. These five proteins collectively predicted resolution of critical illness within 7 days in the STAAMP dataset with an AUC of 0.81 (Figure 2D). Of note, this group of patients had more resolvers (*n* = 46) than non-resolvers (*n* = 18).

### 3.3. Multi-Omic Markers from Three Omic Data Layers Predict 30-Day Survival

**Figure 3:**
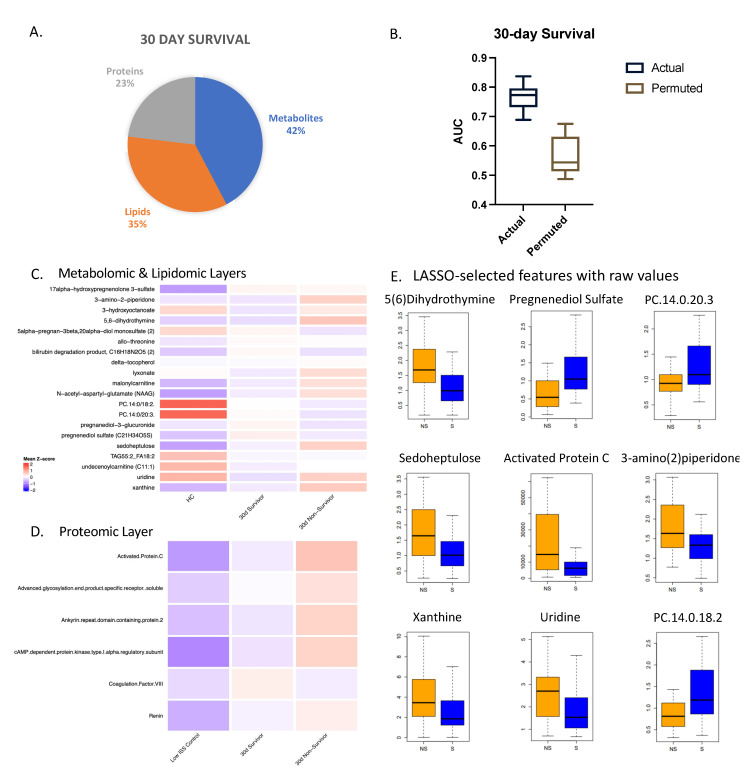
Multi-omic markers predict 30-day survival. (**A**) Breakdown of markers from LASSO model for 30-day survival with a total of 26 features. (**B**) Actual and permuted LASSO AUC graph with an AUC of 0.77. (**C**) Heatmaps of metabolomic and lipidomic layers comparing healthy controls (HC), 30-day survivors, and 30-day non-survivors at the 0 h timepoint, normalized and scaled. (**D**) Numbers of subjects in each group: HC (*n* = 17), 30-day survivors (*n* = 55), 30-day non-survivors (*n* = 57). (**E**) Box plots of LASSO-selected features that are significantly different between survivors and non-survivors with raw value measurement.

For 30-day survival, features identified from the combined multi-omic database were the most predictive of outcome with an AUC of 0.77 (Figure 3). LASSO identified a minimal subset of 26 multi-omic markers (11 metabolites, 9 lipids, 6 proteins) listed in Table S4 from approximately 1500 markers. The proteomic, lipidomic, and metabolomic individual layers yielded AUCs less than 0.72. After carrying out a Benjamini–Hochberg correction for multiple testing, nine of these features measured at admission were found to be significantly different between survivors and non-survivors with an adjusted *p*-value < 0.03.

Relative values of all the selected features were projected for each layer in heatmaps (Figure 3C,D) comparing controls, 30-day survivors, and 30-day non-survivors. Most of the metabolites were higher in non-survivors except for lipids, which were consistently lower in non-survivors, consistent with our previous studies [11,12]. Several proteins related to coagulation were selected as part of the model. Activated protein C was found to be significantly higher in 30-day non-survivors, while coagulation factor VIII showed the opposite pattern.

**Figure 4:**
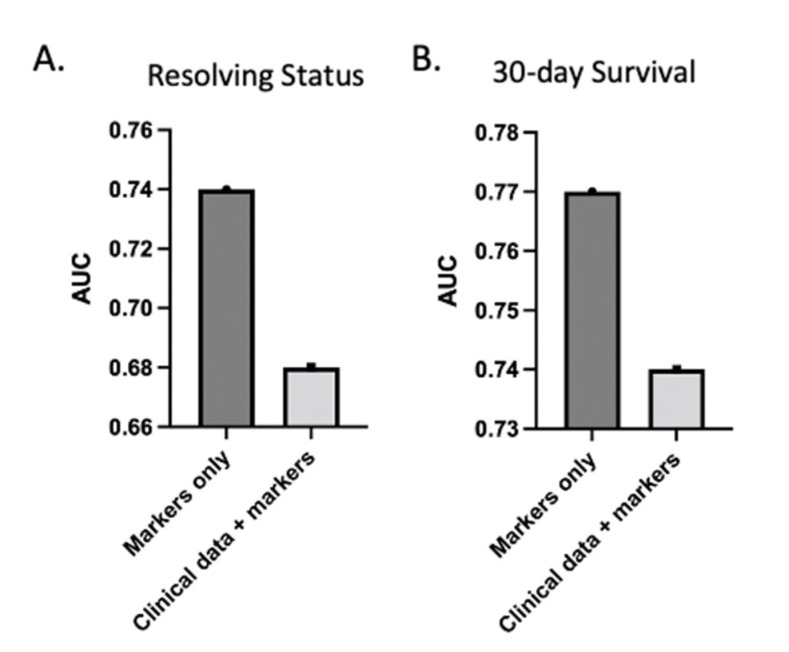
Clinical data do not increase AUC of predictive models. AUC of LASSO model with both clinical variables and markers decreased relative to model with markers alone for both resolving status (**A**) and 30-day survival (**B**). Clinical variables include admission ISS, age, BMI, systolic blood pressure, diastolic blood pressure, mean arterial pressure, heart rate, shock index, lactate, and bicarbonate levels.

To assess the prognostic capability of admission and multi-omic markers relative to clinical variables, we ran the same models but incorporated clinical variables into the model, including age, ISS, BMI, and other factors (Figure 4). ISS and BMI were part of the selected features; however, the AUC dropped for both resolution from critical illness and 30-day survival compared to the models with multi-omic markers only. ISS alone yielded an AUC of 0.68. This indicates that the clinical variables did not add further value to the predictive potential of these biomarkers for this outcome group. We note that ISS is not typically calculated at admission, as the full extent of injuries is often not known at that stage.

### 3.4. Unique Admission Multi-Omic Markers Predict 30-Day Survival in Patients with Traumatic Brain Injury

**Figure 5:**
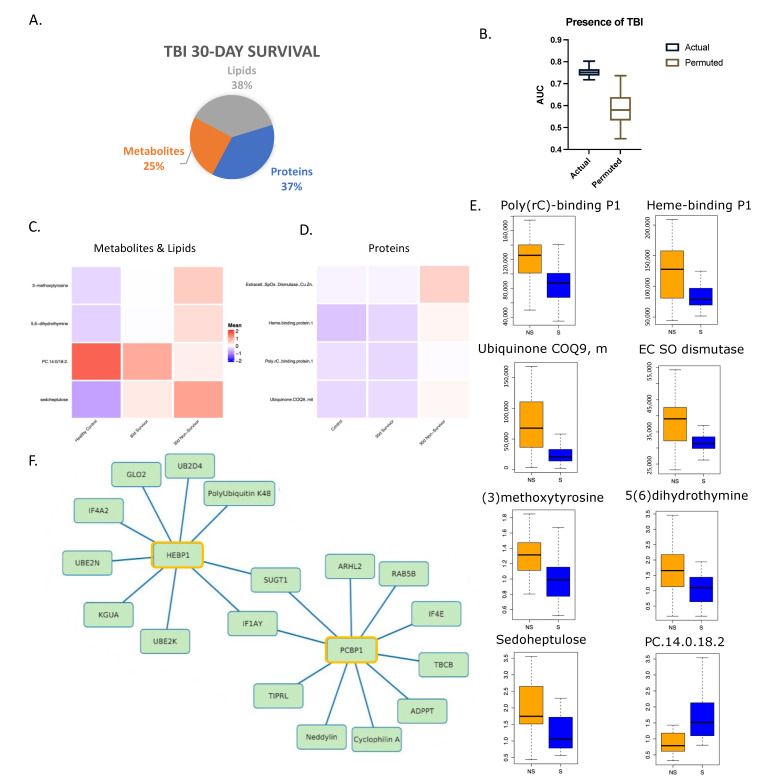
Multi-omic markers predict survival in trauma patients with traumatic brain injury. (**A**) Breakdown of markers from LASSO model output with a total of 8 features. (**B**) Actual and permuted LASSO AUC graph with an AUC of 0.75. (**C**) Heatmap of metabolomic and lipidomic selected features looking at 30-day survival in patients with TBI only and healthy controls. Healthy controls *n* = 17, 30-day survivors *n* = 27, non-survivors *n* = 37. (**D**) Heatmap of proteomic features as in (**C**). Low ISS controls *n* = 29, 30-day survivors *n* = 21, non-survivors *n* = 28. (**E**) Box plots of LASSO-selected features that are significantly different between 30-day survivors and non-survivors with raw value measurement. (**F**) Correlation network between selected features and proteomic database at Spearman’s r = 0.70 showing direct associations (selected feature outlined in yellow).

A combination of eight admission biomarkers was selected by LASSO analysis from the multi-omic dataset for the prediction of 30-day survival in patients with CT-confirmed TBI. As shown in Figure 5A, selected features yielded an AUC of 0.75 (Figure 5B and Table S5). The individual layers and a combination of these layers all yielded AUCs below 0.72. We compared the relative levels of these markers in the heatmaps (Figure 5C,D) with healthy controls (*n* = 17) and low ISS controls over time (*n* = 29). For metabolomics and lipidomics, 27 survivors and 37 non-survivors were included, while 21 and 28 were included for proteomics, respectively. All the biomarkers were significantly higher (*p* < 0.05) in non-survivors except for PC.14:0/18:2 (raw value plotted in Figure 5E), a phosphatidylcholine phospholipid.

A correlation network analysis revealed several strong associations among these LASSO-selected features with other proteins (Figure S3). HEBP1, or heme-binding protein 1 (Figure 5F), is thought to bind free toxic porphyrinogens present in the cell as well as promote chemotaxis in monocytes and dendritic cells [26,27]. It is associated with several ubiquitin proteins related to protein degradation and DNA repair. Hydroxyacylglutathione hydrolase, which is vital for the production of D-lactic acid [23,28], was also highly correlated to HEBP1.

Two proteins, eukaryotic translation initiation factor 1A (IF1AY) and protein SGT1 homolog (SUGT1), were correlated to two of the selected features, namely HEBP1 and Poly(rC)-binding protein 1 (PCBP1). IF1AY and SUGT1 both have functions related to protein biosynthesis and degradation [23,29]. PCBP1, a single-stranded nucleic acid binding protein, has a known role in initiation of viral RNA replication and translation [23,29]. It is associated with several proteins shown in Figure 5F related to the regulation of DNA damage, recovery from replicative DNA stress, vesicular transport, and modification of post-translational proteins [23,29]. Peptidyl-prolyl cis-trans isomerase A (cyclophilin A) has strong chemotactic effects on leukocytes as well as a role in initiating a cascade that activates the MAPK/ERK pathway. It also exerts a pro-inflammatory effect on endothelial cells through NF-kappa-B and MAP-kinases [23].

Taken together, these analyses show that the addition of TBI to the injury complex impacts the features that predict survival.

### 3.5. Gene Set Enrichment Analysis

Gene set enrichment analysis (GSEA) was carried out between the outcome subgroups (survivors vs. non-survivors and resolvers vs. non-resolvers, separately). Supplementary Tables S6–S8 contain the genes with the strongest positive and negative scores that are associated with each outcome analysis. A positive score indicated an enriched gene in the first phenotype (30-day survivors, resolvers, TBI survivors), while a negative score indicated enriched genes in the second phenotype (30-day non-survivors, non-resolvers, and TBI non-survivors).

The “hallmark coagulation” gene set shown in Figure S4 was enriched in patients with more favorable outcomes, including 30-day survivors and resolvers, suggesting the loss or consumption of many coagulation factors in patients with worse outcomes. This gene set is comprised of 138 genes involved in the blood-coagulation system and platelet function [30]. Consistent with the LASSO analysis, the vitamin-K-dependent inactive form of protein C was significantly (*p* < 0.05) higher in resolvers, and coagulation factor VIII was highest in 30-day survivors. On the other hand, activated protein C (ApC) was significantly higher in both 30-day non-survivors and non-resolving patients, as shown by the tests of significance from the LASSO-selected features.

We also identified the “hallmark interferon-alpha response” gene set, comprised of 97 genes that are upregulated in response to alpha interferons [31] in 30-day non-survivors and non-resolving patients. This gene set includes proteins related to cytokines and growth factors, transcription factors, cell differentiation markers, and protein kinases.

## 4. Discussion

This study was undertaken to identify predictive biomarkers using high-dimensional multi-omic datasets derived from admission blood draws. By utilizing the standard-of-care arm patients from two recent interventional trials we were able to take advantage of patients enrolled across multiple institutions to better understand the features of severely injured humans. A multivariate machine learning model identified the combination of admission omics features that were most predictive for death within 30 days or resolution of critical illness. A consistent feature from both analyses was higher levels of activated protein C in patients destined for adverse outcomes. Gene enrichment analysis also strongly identified components of coagulation as correlative with patient outcomes. The inclusion of TBI in the injury complex altered the prognostic markers, indicating the polytrauma patients with TBI are likely to require unique models to predict outcomes.

Trauma management has become standardized in order to have consistent and systematic care for patients by following guidelines such as the Advanced Trauma Life Support [32,33]. However, there is still a need to identify admission biomarkers that are highly predictive of adverse outcomes in order to prioritize and intensify care [34,35,36]. We recently reported a circulating leukocyte transcriptomic classifier that accurately separated trauma patients into those with rapid vs. slow resolution based on transcriptomic patterns assessed at <12 h [37]. Raymond et al. showed that a NanoString-based assessment of 63 genes in whole blood measured at 24 h yielded an AUC of 0.84 [38]. Salve et al. showed that admission serum copeptin predicted ISS > 15 and need for hospital admission [39]. Other studies examined base deficit and blood lactate levels during early triage to differentiate major from minor trauma and as prognostic markers [40,41]. We report here the first effort to use high-dimensional multi-omic datasets to identify admission-based biomarkers for adverse outcomes. Of note, these datasets include a large number of immune mediators and metabolites previously studied in human trauma, including inflammatory mediators and metabolites such as lactate and succinate. However, only the chemokine IL-8 was identified by our machine learning analysis. This supports the notion that yet unstudied biomarkers could have great utility in trauma. It is notable that the modeling yielded AUCs in the high 0.7 range. Furthermore, the combinations of variables that were most predictive were dependent on the outcome of interest. This raises the possibility that predictive panels may need to be adjusted to capture both mortality and resolution from critical illness. The inclusion of other variables including patient demographics and clinical variables would be likely to increase the sensitivity but did not increase the AUC of any of the models.

The GSEA and correlation network analysis yielded novel results with regards to biological pathways related to traumatic injury. The MYC target genes are reported to be involved in a large array of biological processes and have been studied in animals as regulators of energy metabolism in response to renal injury, particularly facilitating glycolysis [42]. Our previous analysis demonstrated a “systemic storm” early after injury that included a massive release of molecules, many of which are energy metabolites, into the circulation. Notably, this release was more extensive in non-survivors [10]. Another selected feature specific to TBI 30-day survivor outcome was sedoheptulose, which controls the interplay between glycolysis and the pentose phosphate pathway by functioning as a biological switch [43].

In patients with worse outcomes (non-survivors and non-resolvers), heme metabolism was enriched prominently compared to survivors and resolvers. The literature includes reports studying the relation between heme-related products and injury severity, especially in association with different arms of the immune system and brain recovery after stroke and TBI [44,45,46,47]. Heme-binding P1 was a selected feature in predicting 30-day survival in TBI patients as well. Furthermore, multiple studies document the detrimental role of the interferon response in the context of brain injury [48,49]. Enriched alpha interferon response pathways were noted in 30-day non-survivors with patients who have confirmed TBI and in the group that includes patients with and without TBI.

The strengths of this study include a tightly controlled standard-care patient cohorts from the PAMPer clinical trial, along with available multi-omic databases. We included cross prediction and validation in an unrelated group of patients from another trauma clinical trial (STAAMP). The limitations include the inability to establish causality. The metabolomic layer uses qualitative ultra-performance liquid chromatography for measurement and the results should be validated with quantitative assays. Ultimately, the use of many of these biomarkers in the clinical setting would require validated assays. However, our findings can serve as a guide and resource to identify sets of biomarkers that combine predictive power and ease of measurement.

## Figures and Tables

**Table 1:** Demographic characteristics of patients used to train the LASSO models based on presence of traumatic brain injury (TBI).

PAMPer Cohort	TBI	No TBI	*p*-Value
**N**	64	48	
**Age (Average)**	45.9	46.2	
**Gender**			>0.05
**Males**	53	35	
**Females**	11	13	
**Outcome**			<0.05
**Resolver**	8	17	
**Non-resolver**	34	16	
**Early non-survivor**	22	15	
**ISS**			<0.05
**Mild (ISS < 15)**	4	13	
**Moderate (ISS 16–25)**	12	17	
**Severe (ISS > 25)**	48	18	
**Treatment**			<0.05
**Standard of care**	49	35	
**Experimental**	38	27	
**Type of Injury**			<0.05
**Blunt**	64	36	
**Penetrating**	0	11	
**Both**	0	1	
**30-day Survival**			>0.05
**Yes**	27	28	
**No**	37	20	
**Shock**			0.29
**Yes**	34	26	
**No**	30	22	

**Table 2:** LASSO model AUC and accuracies for each group including validation cohort.

LASSO MODEL	AREA UNDER THE ROC CURVE	ACCURACY
**30-DAY SURVIVAL**	0.77	70%
**RESOLVING STATUS**	0.74	70%
**VALIDATION COHORT FOR RESOLVING STATUS**	0.81	78%
**30-DAY SURVIVAL IN TBI PATIENTS**	0.75	76%

## Data Availability

The data presented in this study are openly available in https://data.mendeley.com/datasets/vt8nhp2y2t/1 (accessed on 1 June 2022).

## Supplementary Materials

The following supporting information can be downloaded at: https://www.mdpi.com/article/10.3390/metabo12090774/s1, Figure S1: Flow chart of methods used for feature selection using LASSO; Figure S2: Correlation network analysis between LASSO-model selected features for predicting persistent critical illness in trauma patients (Spearman’s r > 0.70); Figure S3: Correlation network analysis between LASSO-model selected features for predicting 30-day survival in trauma patients (Spearman’s r > 0.70); Figure S4: Top phenotype gene set enrichment analysis in each group; Figure S5: Correlation network analysis between LASSO-model selected features for predicting traumatic brain injury in trauma patients (Spearman’s r > 0.70). Table S1: AUC’s of LASSO runs done for each outcome group; Table S2: Selected features for resolving status; Table S3: List of protein names for correlation networks; Table S4: Selected features for 30-day survival; Table S5: Selected features for 30-day survival in TBI; Table S6: Ranked Gene List between 30-day survivors vs. non-survivors (GSEA); Table S7: Ranked gene list between resolvers and non-resolvers (GSEA); Table S8: Ranked Gene List between TBI vs. NBI patients (GSEA).
