# Supplementary material for: Multi-Omic Admission-Based Prognostic Biomarkers Identified by Machine Learning Algorithms Predict Patient Recovery and 30-Day Survival in Trauma Patients
Source: Metabolites. 2022 Aug 23;12(9):774. doi: 10.3390/metabo12090774 (PMC9500723; doi:10.3390/metabo12090774)
Supplement: Supplementary file 1 [file metabolites-12-00774-s001.zip › metabolites-1831225-supplementary.pdf]

Supplementary figures and tables

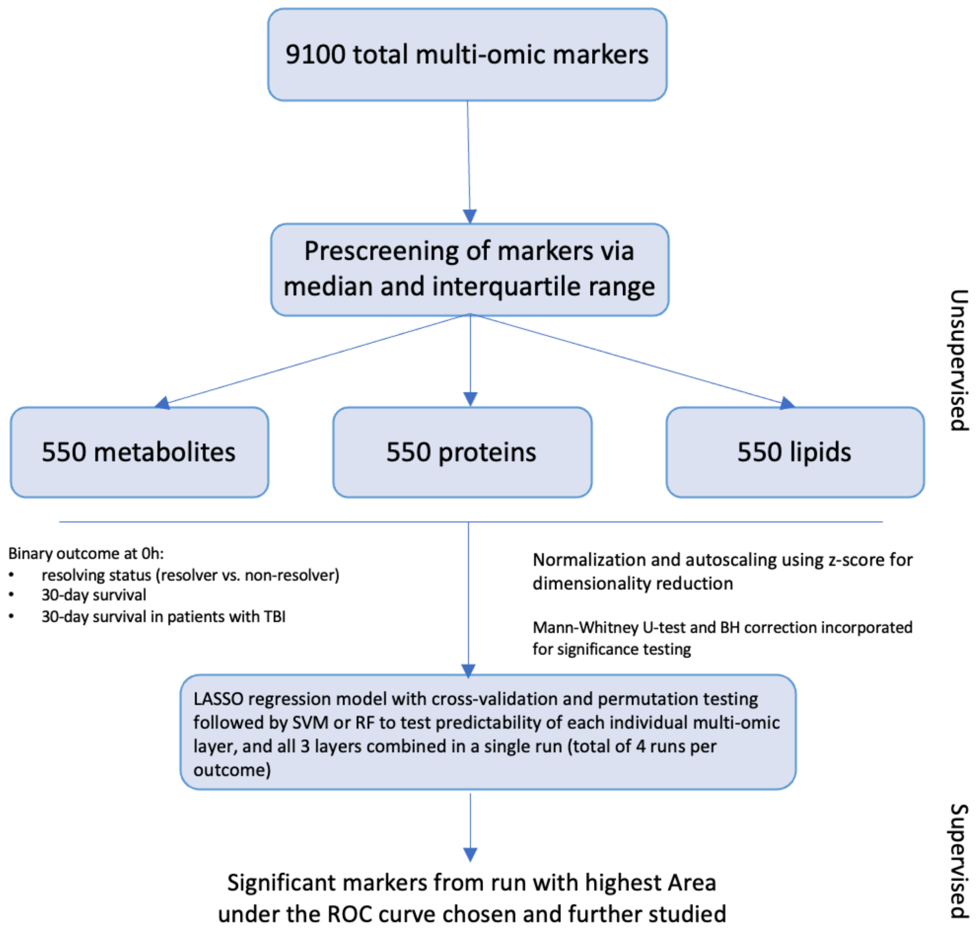

Figure S1 Flow chart of methods used for feature selection using LASSO.

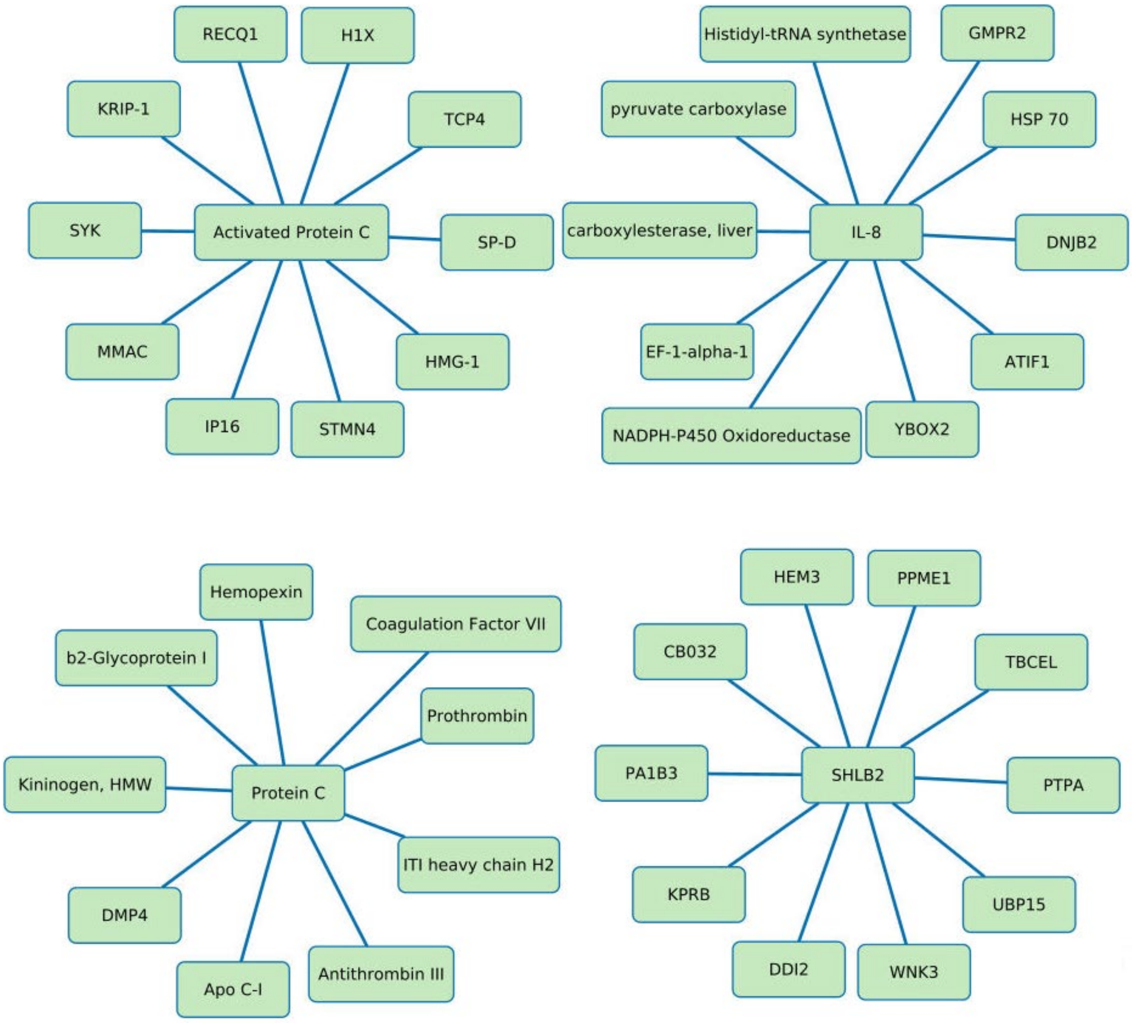

Figure S2. Correlation network analysis between LASSO-model selected features for predicting persistent critical illness in trauma patients (Spearman's r >.70)

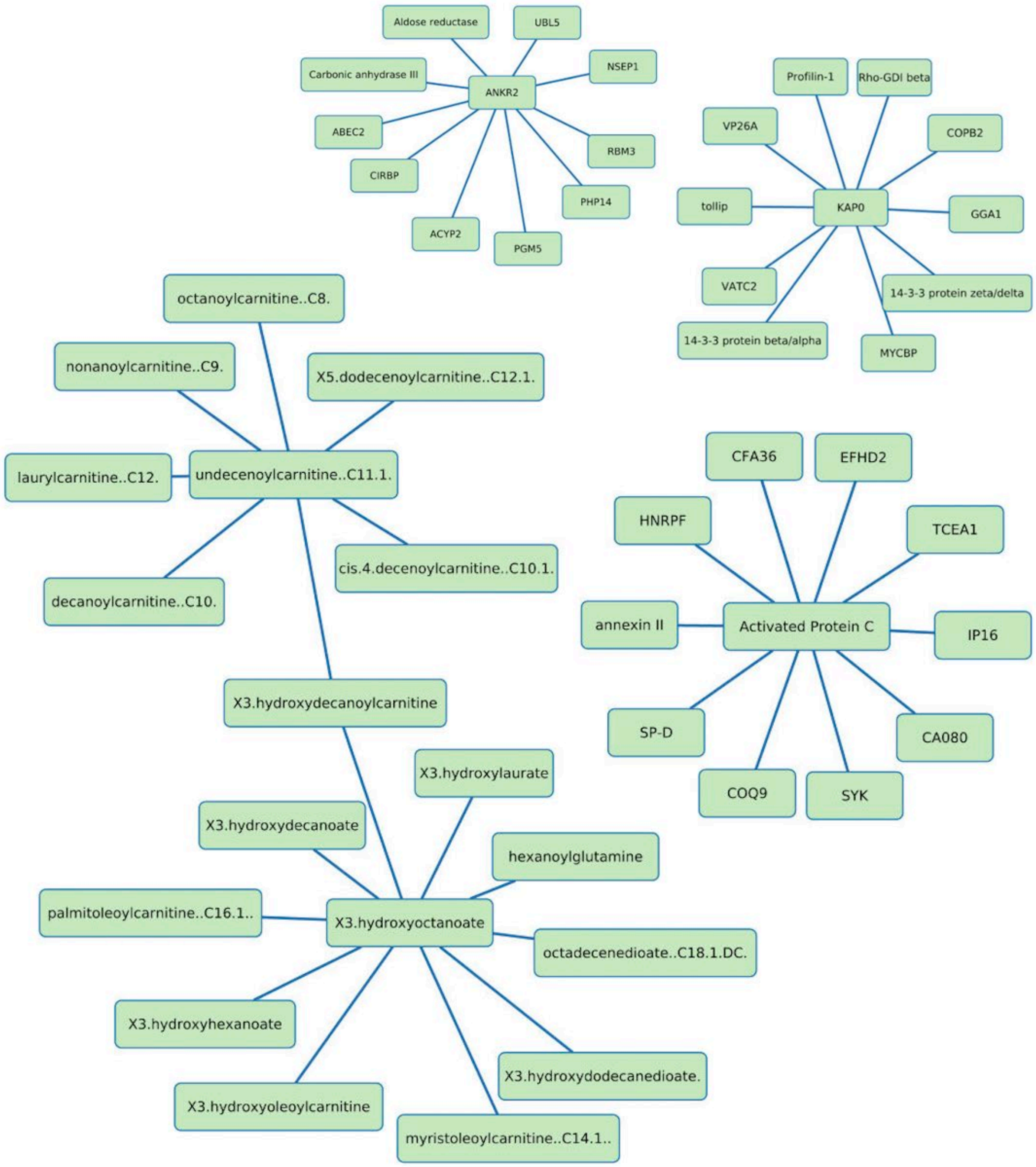

Figure S3. Correlation network analysis between LASSO-model selected features for predicting 30-day survival in trauma patients (Spearman's r >.70)

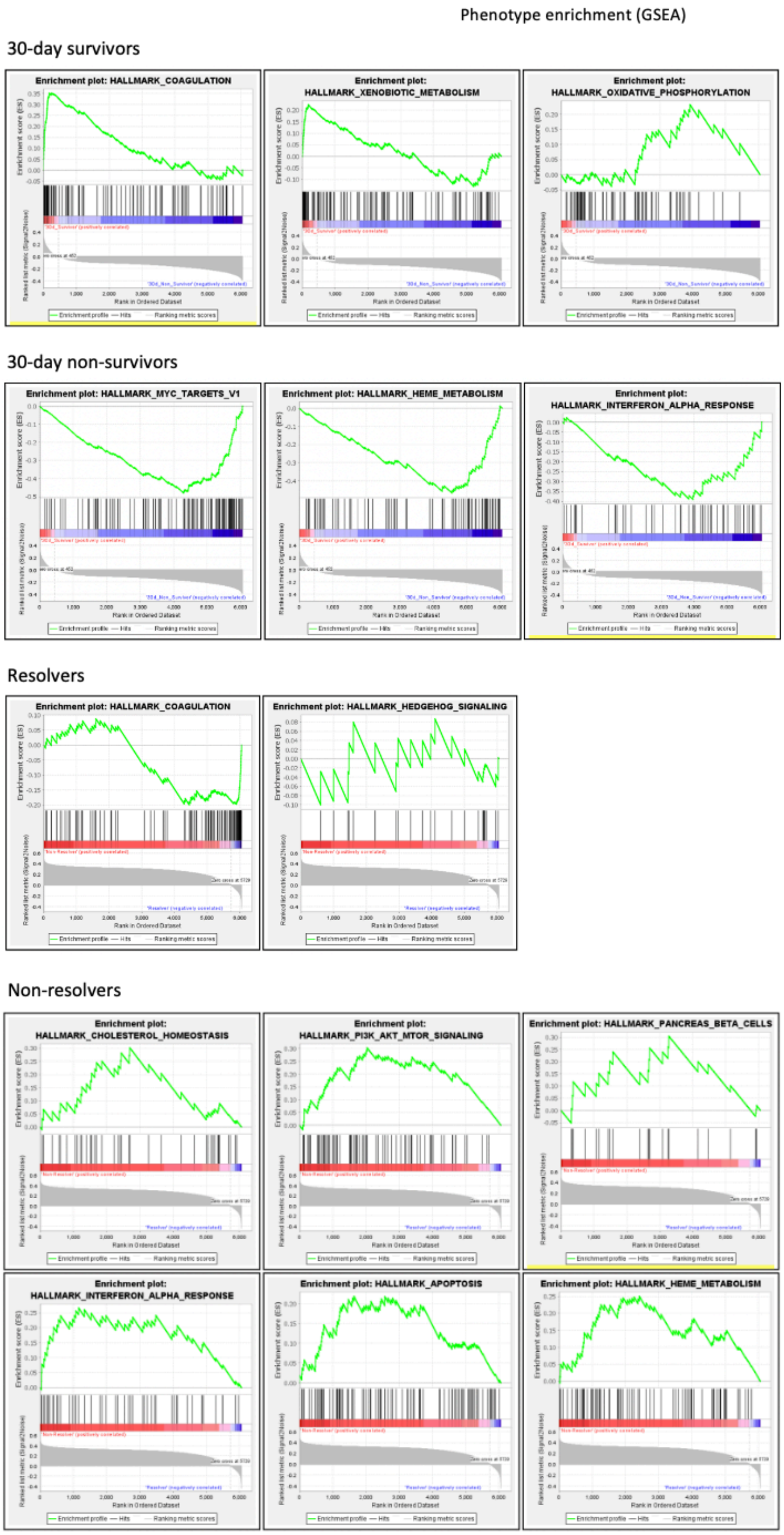

Figure S4. Top phenotype gene set enrichment analysis in each group (A. & B. 30-day survivors and non-survivors, C. & D. resolvers and non-resolvers). Y-axis is the enrichment score (ES) which indicates to what degree a gene set is enriched in a given group when compared to the other group. X-axis demonstrates “Rank in ordered dataset” which measures a gene’s correlation with a specific phenotype. A positive value demonstrates a correlation with the first phenotype (30-day survivors in group 1, resolvers in group 2), and a negative value demonstrates a correlation with the second phenotype (30-day non-survivors in group 1, non-resolvers in group 2).

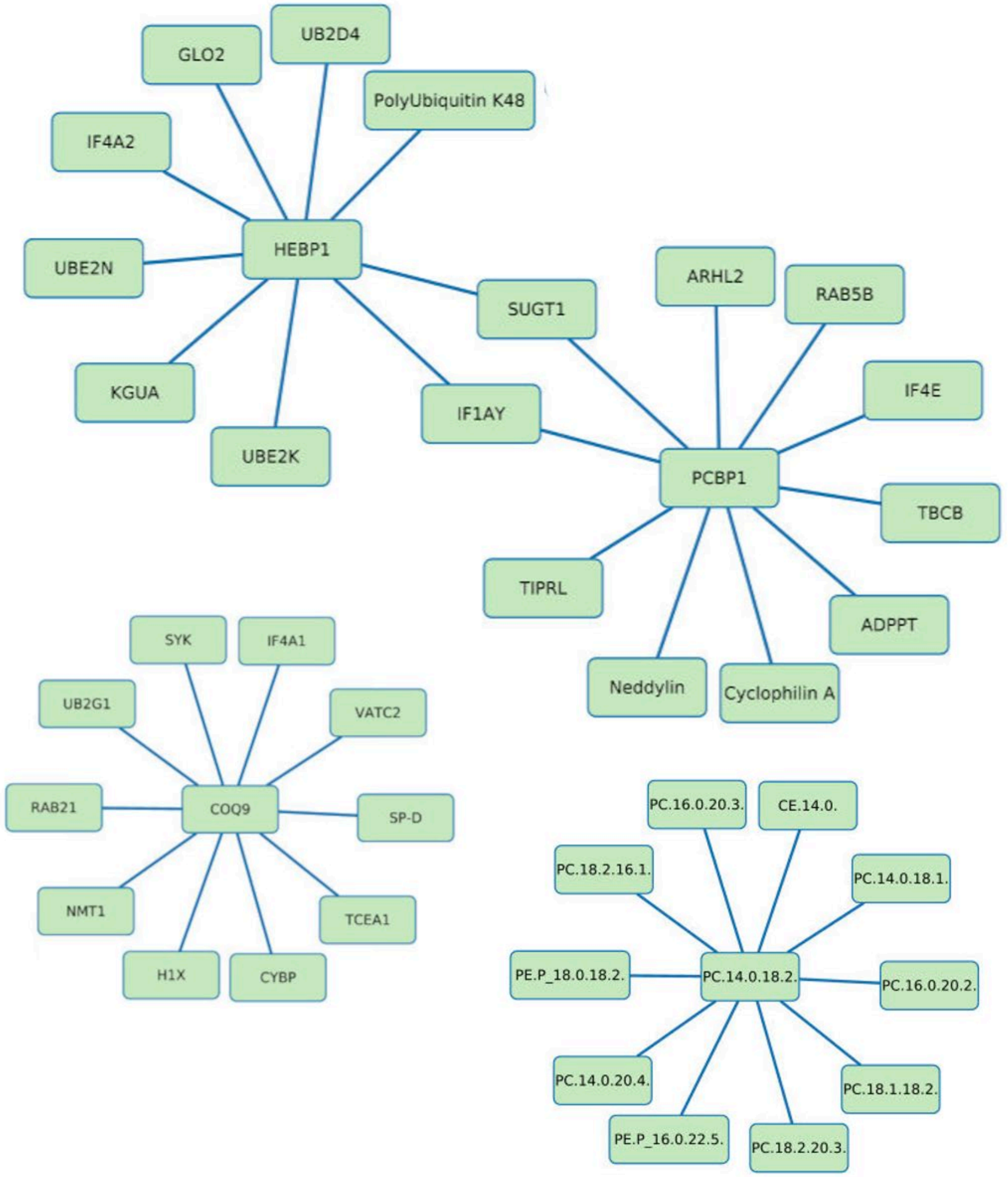

Figure S5. Correlation network analysis between LASSO-model selected features for predicting traumatic brain injury in trauma patients (Spearman's r >.70).

Table S1. AUC's of LASSO runs done for each outcome group (4 runs each, proteomic layer, metabolomic layer, lipidomic layer, and a combined layer from the previous 3).

	AUC
**RESOLVING STATUS**	
**PROTEOMIC**	0.74
**LIPIDOMICS**	0.62
**METABOLOMICS**	0.65
**COMBINED**	0.57
**30-DAY SURVIVAL**	
**COMBINED**	0.77
**PROTEOMICS**	0.63
**LIPIDOMICS**	0.63
**METABOLOMICS**	0.7
**30-DAY SURVIVAL/TBI**	
**COMBINED**	0.75
**PROTEOMICS**	0.66
**LIPIDOMICS**	0.55
**METABOLOMICS**	0.72

Table S2. Selected features for resolving status.

SELECTED FEATURES FOR RESOLVING STATUS	CLASS	PATHWAY	PVALUE	BH CORRECTED P-VALUE
Beta-microseminoprotein	Protein	Immunoglobulin Binding Family	0.0000711	0.000284
Interleukin-8	Protein	Chemokine	0.0000649	0.000284
Endophilin-B2	Protein	Endophilin B Family	0.00392807	0.010691
Activated Protein C	Protein	Coagulation	0.00534534	0.010691
Vitamin K-dependent protein C	Protein	Coagulation	0.01486754	0.014868

Table S3. List of protein names for correlation networks.

PROTEIN TARGET NAME	PROTEIN FULL NAME
**ACTIVATED PROTEIN C**	Activated Protein C
**ADPPT**	L-aminoadipate-semialdehyde dehydrogenase-phosphopantetheinyl transferase
**ARHL2**	Poly(ADP-ribose) glycohydrolase ARH3
**CB032**	CB1 cannabinoid receptor-interacting protein 1
**CYCLOPHILIN A**	Peptidyl-prolyl cis-trans isomerase A
**DDI2**	Protein DDI1 homolog 2
**GLO2**	Hydroxyacylglutathione hydrolase, mitochondrial
**H1X**	Histone H1x
**HEBP1**	Heme-binding protein 1
**HEM3**	Porphobilinogen deaminase
**HMG-1**	High mobility group protein B1
**IF1AY**	Eukaryotic translation initiation factor 1A, Y-chromosomal
**IF4A2**	Eukaryotic initiation factor 4A-II
**IF4E**	Eukaryotic translation initiation factor 4E
**IP16**	Gamma-interferon-inducible protein 16
**KGUA**	Guanylate kinase
**KPRB**	Phosphoribosyl pyrophosphate synthase-associated protein 2

**KRIP-1**	Transcription intermediary factor 1-beta
**MMAC**	Methylmalonic aciduria and homocystinuria type C protein
**NEDDYLIN**	Ubiquitin-like protein Nedd8
**PA1B3**	Platelet-activating factor acetylhydrolase IB subunit gamma
**PCBP1**	Poly(rC)-binding protein 1
**POLYUBIQUITIN K48**	PolyUbiquitin K48-linked
**PPME1**	Protein phosphatase methylesterase 1
**PTPA**	Serine/threonine-protein phosphatase 2A regulatory subunit B'
**RAB5B**	Ras-related protein Rab-5B
**RECQ1**	ATP-dependent DNA helicase Q1
**SHLB2**	Endophilin-B2
**SP-D**	Pulmonary surfactant-associated protein D
**STMN4**	Stathmin-4
**SUGT1**	Protein SGT1 homolog
**SYK**	Lysine--tRNA ligase
**TBCB**	Tubulin-folding cofactor B
**TBCEL**	Tubulin-specific chaperone cofactor E-like protein
**TCP4**	Activated RNA polymerase II transcriptional coactivator p15
**TIPRL**	TIP41-like protein
**UB2D4**	Ubiquitin-conjugating enzyme E2 D4
**UBE2K**	Ubiquitin-conjugating enzyme E2 K
**UBE2N**	Ubiquitin-conjugating enzyme E2 N
**UBP15**	Ubiquitin carboxyl-terminal hydrolase 15
**WNK3**	Serine/threonine-protein kinase WNK3

Table S4. Selected features for 30-day survival.

SELECTED FEATURES FOR 30-DAY SURVIVAL	CLASS	PATHWAY	P-VALUE	BH CORRECTED P-VALUE
**5(6)DIHYDROTHYMINE**	Metabolite	Pyrimidine Metabolism	0.00000502	0.000131
**PREGNENEDIOL SULFATE (C21H34O5S)**	Lipid	Pregnenolone Steroids	0.000202	0.005055
**PC.14.0.20.3**	Lipid	Phosphatidylcholine	0.0003	0.007196
**SEDOHEPTULOSE**	Metabolite	Monosaccharide	0.000332	0.00764
**ACTIVATED PROTEIN C**	Protein	Coagulation	0.000858	0.01761
**(3)AMINO(2)PIPERIDONE**	Metabolite	Arginine and Proline Metabolism	0.000847	0.01761
**XANTHINE**	Metabolite	Purine Metabolism	0.000881	0.01761
**URIDINE**	Metabolite	Pyrimidine Metabolism	0.001153	0.021916
**PC.14.0.18.2**	Lipid	Phosphatidylcholine	0.001277	0.022987
**(17)ALPHA-HYDROXYPREGNENOLONE(3)SULFATE**	Lipid	Pregnenolone Steroids	0.003274	0.055664
**N-ACETYL-ASPARTYL-GLUTAMATE (NAAG)**	Metabolite	Peptide Neurotransmitter	0.003896	0.062342
**ANKYRIN REPEAT DOMAIN-CONTAINING PROTEIN 2**	Protein	MARP Family	0.006512	0.09768
**CAMP-DEPENDENT PROTEIN KINASE TYPE I-ALPHA REGULATORY SUBUNIT**	Protein	Kinase	0.012553	0.146847
**(3)HYDROXYOCTANOATE**	Lipid	Fatty Acid, Monohydroxy	0.014685	0.146847
**LYXONATE**	Metabolite	Pentose Metabolism	0.010516	0.146847
**MALONYLCARNITINE**	Lipid	Fatty Acid Synthesis	0.013936	0.146847
**PREGNANEDIOL(3)GLUCURONIDE**	Lipid	Progestin Steroids	0.012484	0.146847

**(5)ALPHA-PREGNAN(3)BETA(20)ALPHA-DIOL-MONOSULFATE(2)**	Lipid	Progestin Steroids	0.027047	0.216374
**DELTA-TOCOPHEROL**	Metabolite	Tocopherol Metabolism	0.026233	0.216374
**ALLO-THREONINE**	Metabolite	Glycine, Serine and Threonine Metabolism	0.088166	0.617164
**COAGULATION FACTOR VIII**	Protein	Coagulation	0.254972	0.763278
**RENIN**	Protein	Renin Angiotensin System	0.339788	0.763278
**ADVANCED GLYCOSYLATION END PRODUCT-SPECIFIC RECEPTOR, SOLUBLE**	Protein	Cell Surface Molecule	0.411155	0.763278
**BILIRUBIN DEGRADATION PRODUCT (C16H18N2O5.2)**	Metabolite	Bilirubin Degradation	0.270903	0.763278
**UNDECENOYLCARNITINE(C11.1)**	Metabolite	Leucine, Isoleucine and Valine Metabolism	0.132665	0.763278
**TAG55.5 FA18(2)**	Lipid	Triacylglycerol	0.763278	0.763278

Table S5. Selected features for 30-day survival in TBI.

SELECTED FEATURES FOR PRESENCE OF TBI	CLASS	PATHWAY	P-VALUE	BH CORRECTED P-VALUE
N-acetyl-aspartyl-glutamate (NAAG)	Metabolite	Peptide Neurotransmitter	0.0000178	0.000178
Aldose reductase	Protein	NADPH-dependant Oxireductase	0.000021	0.000189
Oxindolylalanine	Metabolite	Tryptophan Metabolism	0.0000317	0.000254
Creatine kinase M-type:Creatine kinase B-type heterodimer	Protein	Kinase	0.000144	0.00101
Fatty acid-binding protein, heart	Protein	Lipid-binding protein	0.000919	0.005513
N-palmitoylglycine	Lipid	Fatty Acid Metabolism (Acyl Glycine)	0.002224	0.011122
5-alpha-androstan(3)beta 17beta-diol-disulfate	Lipid	Androgenic Steroid	0.003814	0.013146
PI.18.0.18.2	Lipid	Phosphatidylinositol	0.004382	0.013146
Oxalate ethanedioate	Metabolite	Ascorbate and Aldarate Metabolism	0.047038	0.076462
Trigonelline(N)methylnicotinate	Metabolite	Nicotinate and Nicotinamide Metabolism	0.076462	0.076462

Table S6. Ranked Gene List between 30-day survivors vs. non-survivors (GSEA). A positive value demonstrates a correlation with the first phenotype (30-day survivors in group 1, resolvers in group 2), and a negative value demonstrates a correlation with the second phenotype (30-day non-survivors in group 1, non-resolvers in group 2).

NAME	TITLE	SCORE
**SERPINF2**	serpin family F member 2 [Source:HGNC Symbol;Acc:HGNC:9075]	0.49038604
**PROC**	protein C, inactivator of coagulation factors Va and VIIIa [Source:HGNC Symbol;Acc:HGNC:9451]	0.47138682
**CPB2**	carboxypeptidase B2 [Source:HGNC Symbol;Acc:HGNC:2300]	0.3814521
**ADAMTS13**	ADAM metallopeptidase with thrombospondin type 1 motif 13 [Source:HGNC Symbol;Acc:HGNC:1366]	0.35133052
**IGFALS**	insulin like growth factor binding protein acid labile subunit [Source:HGNC Symbol;Acc:HGNC:5468]	0.34175417
**PZP**	PZP alpha-2-macroglobulin like [Source:HGNC Symbol;Acc:HGNC:9750]	0.33677304
**PLA2G12B**	phospholipase A2 group XIIB [Source:HGNC Symbol;Acc:HGNC:18555]	0.33042234
**BTD**	biotinidase [Source:HGNC Symbol;Acc:HGNC:1122]	0.3218178
**F9**	coagulation factor IX [Source:HGNC Symbol;Acc:HGNC:3551]	0.3175016
**SERPINA4**	serpin family A member 4 [Source:HGNC Symbol;Acc:HGNC:8948]	0.31455392
**F11**	coagulation factor XI [Source:HGNC Symbol;Acc:HGNC:3529]	0.31378555
**SERPIND1**	serpin family D member 1 [Source:HGNC Symbol;Acc:HGNC:4838]	0.28808224
**NCMAP**	non-compact myelin associated protein [Source:HGNC Symbol;Acc:HGNC:29332]	0.28789428
**IZUMO4**	IZUMO family member 4 [Source:HGNC Symbol;Acc:HGNC:26950]	0.28626233
**F13A1**	coagulation factor XIII A chain [Source:HGNC Symbol;Acc:HGNC:3531]	0.28528827
**IGFBP3**	insulin like growth factor binding protein 3 [Source:HGNC Symbol;Acc:HGNC:5472]	0.28493634
**MAP2K4**	mitogen-activated protein kinase kinase 4 [Source:HGNC Symbol;Acc:HGNC:6844]	0.28217614
**AZGP1**	alpha-2-glycoprotein 1, zinc-binding [Source:HGNC Symbol;Acc:HGNC:910]	0.28040224
**RBP4**	retinol binding protein 4 [Source:HGNC Symbol;Acc:HGNC:9922]	0.27747256
**IL1RAP**	interleukin 1 receptor accessory protein [Source:HGNC Symbol;Acc:HGNC:5995]	0.27242404
**F13B**	coagulation factor XIII B chain [Source:HGNC Symbol;Acc:HGNC:3534]	0.27198485
**CAMP**	cathelicidin antimicrobial peptide [Source:HGNC Symbol;Acc:HGNC:1472]	0.2719579
**SERPINA5**	serpin family A member 5 [Source:HGNC Symbol;Acc:HGNC:8723]	0.2696333
**ARF1**	ADP ribosylation factor 1 [Source:HGNC Symbol;Acc:HGNC:652]	-0.38243207
**INPP5D**	inositol polyphosphate-5-phosphatase D [Source:HGNC Symbol;Acc:HGNC:6079]	-0.38251507
**RFK**	riboflavin kinase [Source:HGNC Symbol;Acc:HGNC:30324]	-0.38626677
**MZB1**	marginal zone B and B1 cell specific protein [Source:HGNC Symbol;Acc:HGNC:30125]	-0.38899603
**TTC9**	tetratricopeptide repeat domain 9 [Source:HGNC Symbol;Acc:HGNC:20267]	-0.3910564
**EIF4A1**	eukaryotic translation initiation factor 4A1 [Source:HGNC Symbol;Acc:HGNC:3282]	-0.39504117
**APLP2**	amyloid beta precursor like protein 2 [Source:HGNC Symbol;Acc:HGNC:598]	-0.39616615
**GZMK**	granzyme K [Source:HGNC Symbol;Acc:HGNC:4711]	-0.39852026
**CPLX1**	complexin 1 [Source:HGNC Symbol;Acc:HGNC:2309]	-0.40014836
**CHMP1A**	charged multivesicular body protein 1A [Source:HGNC Symbol;Acc:HGNC:8740]	-0.4004056
**GPC3**	glypican 3 [Source:HGNC Symbol;Acc:HGNC:4451]	-0.40311554
**ISG15**	ISG15 ubiquitin like modifier [Source:HGNC Symbol;Acc:HGNC:4053]	-0.4225093
**PCNA**	proliferating cell nuclear antigen [Source:HGNC Symbol;Acc:HGNC:8729]	-0.4248515
**SMOC1**	SPARC related modular calcium binding 1 [Source:HGNC Symbol;Acc:HGNC:20318]	-0.4275326
**SLIT2**	slit guidance ligand 2 [Source:HGNC Symbol;Acc:HGNC:11086]	-0.4275852
**A2M**	alpha-2-macroglobulin [Source:HGNC Symbol;Acc:HGNC:7]	-0.43045333
**CPLX2**	complexin 2 [Source:HGNC Symbol;Acc:HGNC:2310]	-0.45341763
**SMOC2**	SPARC related modular calcium binding 2 [Source:HGNC Symbol;Acc:HGNC:20323]	-0.4623694

Table S7. Ranked gene list between resolvers and non-resolvers (GSEA).

NAME	TITLE	SCORE
**CXCL8**	C-X-C motif chemokine ligand 8 [Source:HGNC Symbol;Acc:HGNC:6025]	0.6299099
**MSMB**	microseminoprotein beta [Source:HGNC Symbol;Acc:HGNC:7372]	0.60551965
**ALDH3A1**	aldehyde dehydrogenase 3 family member A1 [Source:HGNC Symbol;Acc:HGNC:405]	0.59096164
**PPT1**	palmitoyl-protein thioesterase 1 [Source:HGNC Symbol;Acc:HGNC:9325]	0.5423089
**GALNT14**	polypeptide N-acetylgalactosaminyltransferase 14 [Source:HGNC Symbol;Acc:HGNC:22946]	0.5222981
**S100A6**	S100 calcium binding protein A6 [Source:HGNC Symbol;Acc:HGNC:10496]	0.49666965
**TMOD1**	tropomodulin 1 [Source:HGNC Symbol;Acc:HGNC:11871]	0.48645607
**CD274**	CD274 molecule [Source:HGNC Symbol;Acc:HGNC:17635]	0.48033306
**EPS8L1**	EPS8 like 1 [Source:HGNC Symbol;Acc:HGNC:21295]	0.48004276
**GDI1**	GDP dissociation inhibitor 1 [Source:HGNC Symbol;Acc:HGNC:4226]	0.4796571
**RILP**	Rab interacting lysosomal protein [Source:HGNC Symbol;Acc:HGNC:30266]	0.47823128
**VSIG2**	V-set and immunoglobulin domain containing 2 [Source:HGNC Symbol;Acc:HGNC:17149]	0.47804528
**CSF3**	colony stimulating factor 3 [Source:HGNC Symbol;Acc:HGNC:2438]	0.47603843
**DHRS11**	dehydrogenase/reductase 11 [Source:HGNC Symbol;Acc:HGNC:28639]	0.4708104
**PA2G4**	proliferation-associated 2G4 [Source:HGNC Symbol;Acc:HGNC:8550]	0.46906778
**CXCL13**	C-X-C motif chemokine ligand 13 [Source:HGNC Symbol;Acc:HGNC:10639]	0.46861416
**ACP6**	acid phosphatase 6, lysophosphatidic [Source:HGNC Symbol;Acc:HGNC:29609]	0.46555108
**FCGR2B**	Fc fragment of IgG receptor IIb [Source:HGNC Symbol;Acc:HGNC:3618]	0.46078134
**DNAJB4**	DnaJ heat shock protein family (Hsp40) member B4 [Source:HGNC Symbol;Acc:HGNC:14886]	0.4603548
**EEF2KMT**	eukaryotic elongation factor 2 lysine methyltransferase [Source:HGNC Symbol;Acc:HGNC:32221]	0.4583485
**JUP**	junction plakoglobin [Source:HGNC Symbol;Acc:HGNC:6207]	0.45665398
**AMPH**	amphiphysin [Source:HGNC Symbol;Acc:HGNC:471]	0.454679
**KRT17**	keratin 17 [Source:HGNC Symbol;Acc:HGNC:6427]	0.44462767
**EIF4EBP3**	eukaryotic translation initiation factor 4E binding protein 3 [Source:HGNC Symbol;Acc:HGNC:3290]	0.44448102
**F9**	coagulation factor IX [Source:HGNC Symbol;Acc:HGNC:3551]	-0.23622549
**CFHR5**	complement factor H related 5 [Source:HGNC Symbol;Acc:HGNC:24668]	-0.23656416
**TMEM70**	transmembrane protein 70 [Source:HGNC Symbol;Acc:HGNC:26050]	-0.23668264
**PLG**	plasminogen [Source:HGNC Symbol;Acc:HGNC:9071]	-0.2384552
**IL20RB**	interleukin 20 receptor subunit beta [Source:HGNC Symbol;Acc:HGNC:6004]	-0.24539113
**FGL1**	fibrinogen like 1 [Source:HGNC Symbol;Acc:HGNC:3695]	-0.25183696
**BTD**	biotinidase [Source:HGNC Symbol;Acc:HGNC:1122]	-0.25381142
**SERPINC1**	serpin family C member 1 [Source:HGNC Symbol;Acc:HGNC:775]	-0.25408944
**ITIH2**	inter-alpha-trypsin inhibitor heavy chain 2 [Source:HGNC Symbol;Acc:HGNC:6167]	-0.2556512
**F7**	coagulation factor VII [Source:HGNC Symbol;Acc:HGNC:3544]	-0.26221678
**AGT**	angiotensinogen [Source:HGNC Symbol;Acc:HGNC:333]	-0.27318233
**GC**	GC vitamin D binding protein [Source:HGNC Symbol;Acc:HGNC:4187]	-0.2844821
**IZUMO4**	IZUMO family member 4 [Source:HGNC Symbol;Acc:HGNC:26950]	-0.32439372
**CPB2**	carboxypeptidase B2 [Source:HGNC Symbol;Acc:HGNC:2300]	-0.33378258
**HP**	haptoglobin [Source:HGNC Symbol;Acc:HGNC:5141]	-0.33667478
**SERPINF2**	serpin family F member 2 [Source:HGNC Symbol;Acc:HGNC:9075]	-0.34237707
**IL1RAP**	interleukin 1 receptor accessory protein [Source:HGNC Symbol;Acc:HGNC:5995]	-0.34765673
**PZP**	PZP alpha-2-macroglobulin like [Source:HGNC Symbol;Acc:HGNC:9750]	-0.399206
**PROC**	protein C, inactivator of coagulation factors Va and VIIIa [Source:HGNC Symbol;Acc:HGNC:9451]	-0.4422778

Table S8. Ranked Gene List between TBI vs. NBI patients (GSEA).

NAME	TITLE	SCORE
**ALDH1A2**	aldehyde dehydrogenase 1 family member A2 [Source:HGNC Symbol;Acc:HGNC:15472]	0.57157636
**CPLX2**	complexin 2 [Source:HGNC Symbol;Acc:HGNC:2310]	0.48933095
**CRABP2**	cellular retinoic acid binding protein 2 [Source:HGNC Symbol;Acc:HGNC:2339]	0.46159774
**HPGDS**	hematopoietic prostaglandin D synthase [Source:HGNC Symbol;Acc:HGNC:17890]	0.44475207
**ELAVL2**	ELAV like RNA binding protein 2 [Source:HGNC Symbol;Acc:HGNC:3313]	0.43242094
**AKR1B1**	aldo-keto reductase family 1 member B [Source:HGNC Symbol;Acc:HGNC:381]	0.41787526
**GALNT14**	polypeptide N-acetylgalactosaminyltransferase 14 [Source:HGNC Symbol;Acc:HGNC:22946]	0.41758662
**MAG**	myelin associated glycoprotein [Source:HGNC Symbol;Acc:HGNC:6783]	0.40778923
**NOVA1**	NOVA alternative splicing regulator 1 [Source:HGNC Symbol;Acc:HGNC:7886]	0.40716144
**NPTXR**	neuronal pentraxin receptor [Source:HGNC Symbol;Acc:HGNC:7954]	0.40158445
**ASB9**	ankyrin repeat and SOCS box containing 9 [Source:HGNC Symbol;Acc:HGNC:17184]	0.3999002
**KLK7**	kallikrein related peptidase 7 [Source:HGNC Symbol;Acc:HGNC:6368]	0.39699575
**MSMB**	microseminoprotein beta [Source:HGNC Symbol;Acc:HGNC:7372]	0.39032647
**USP14**	ubiquitin specific peptidase 14 [Source:HGNC Symbol;Acc:HGNC:12612]	0.389617
**CRHBP**	corticotropin releasing hormone binding protein [Source:HGNC Symbol;Acc:HGNC:2356]	0.38663018
**AGER**	advanced glycosylation end-product specific receptor [Source:HGNC Symbol;Acc:HGNC:320]	0.38479918
**CBR3**	carbonyl reductase 3 [Source:HGNC Symbol;Acc:HGNC:1549]	0.38003492
**CNRIP1**	cannabinoid receptor interacting protein 1 [Source:HGNC Symbol;Acc:HGNC:24546]	0.374988
**NCAN**	neurocan [Source:HGNC Symbol;Acc:HGNC:2465]	0.37451422
**EHD2**	EH domain containing 2 [Source:HGNC Symbol;Acc:HGNC:3243]	0.37395182
**ATP1B1**	ATPase Na+/K+ transporting subunit beta 1 [Source:HGNC Symbol;Acc:HGNC:804]	0.3709674
**PLCD1**	phospholipase C delta 1 [Source:HGNC Symbol;Acc:HGNC:9060]	0.3699091
**YWHAG**	tyrosine 3-monooxygenase/tryptophan 5-monooxygenase activation protein gamma [Source:HGNC Symbol;Acc:HGNC:12852]	0.36614332
**HMBS**	hydroxymethylbilane synthase [Source:HGNC Symbol;Acc:HGNC:4982]	0.3659617
**PHPT1**	phosphohistidine phosphatase 1 [Source:HGNC Symbol;Acc:HGNC:30033]	0.36336586
**ITGA2B**	integrin subunit alpha 2b [Source:HGNC Symbol;Acc:HGNC:6138]	-0.17555647
**GRIK2**	glutamate ionotropic receptor kainate type subunit 2 [Source:HGNC Symbol;Acc:HGNC:4580]	-0.18448848
**ACTN1**	actinin alpha 1 [Source:HGNC Symbol;Acc:HGNC:163]	-0.19070216
**COQ6**	coenzyme Q6, monooxygenase [Source:HGNC Symbol;Acc:HGNC:20233]	-0.20896854
**MMP19**	matrix metallopeptidase 19 [Source:HGNC Symbol;Acc:HGNC:7165]	-0.21055807
**TDGF1**	teratocarcinoma-derived growth factor 1 [Source:HGNC Symbol;Acc:HGNC:11701]	-0.21163894
**CHGA**	chromogranin A [Source:HGNC Symbol;Acc:HGNC:1929]	-0.21710454
**ATP1B4**	ATPase Na+/K+ transporting family member beta 4 [Source:HGNC Symbol;Acc:HGNC:808]	-0.23038776
**FGL1**	fibrinogen like 1 [Source:HGNC Symbol;Acc:HGNC:3695]	-0.27049166
